# Four New Species of *Torula* (Torulaceae, Pleosporales) from Sichuan, China

**DOI:** 10.3390/jof9020150

**Published:** 2023-01-22

**Authors:** Wenhui Tian, Pengwei Su, Yanpeng Chen, Sajeewa S. N. Maharachchikumbura

**Affiliations:** School of Life Science and Technology, Center for Informational Biology, University of Electronic Science and Technology of China, Chengdu 611731, China

**Keywords:** 4 new taxa, asexual morph, Dothideomycetes, phylogeny, taxonomy

## Abstract

*Torula* is an asexual and hyphomycetous genus in the family Torulaceae. *Torula* species are generally saprophytic. They have a worldwide distribution and abound in humid or freshwater habitats. In order to better understand this genus, we carried out several field collections from Sichuan, China. As a result, we obtained nine *Torula* isolates from dead woody substrates in terrestrial and freshwater habitats. Based on a biphasic approach of morphological examination and multi-locus phylogenetic analyses (ITS, SSU, LSU, TEF, RPB2), these collections were identified as belonging to seven *Torula* species. Four of them were new species (*Torula chinensis*, *T. longiconidiophora*, *T. sichuanensis* and *T. submersa*), and the other three belonged to existing species, though one was found for the first time in China (*T. masonii*). Morphological and updated phylogenetic delamination of the new discoveries is also discussed. This study provides further insights into our understanding of wood-based *Torula* species in China.

## 1. Introduction

The family Torulaceae was introduced by Corda with *Torula* as the type [1]. Torulaceae is known only by asexual characteristics of its effuse and powdery colony; mostly immersed mycelia; micro- or macronematous, erect and subcylindrical conidiophores; doliiform to ellipsoid or clavate, cupulate, smooth to verruculose and mono- to polyblastic conidiogenous cells; subcylindrical, phragmosporous, acrogenous, brown, and smooth to verrucose conidia [1,2,3,4,5]. Based on combined ITS and LSU sequence data analyses, Crous et al. [1] revised the family Torulaceae and included the genus *Dendryphion.* Su et al. [2] introduced *Neotorula* from freshwater habitats, and Li et al. [6] reported a *Sporidesmium*-like genus, *Sporidesmioides*, within Torulaceae. Su et al. [5] provided an updated phylogeny for Torulaceae based on the combined ITS, LSU, RPB2, and TEF sequence data analyses and established the monotypic genus *Rostriconidium* from freshwater habitats. Based on ITS and LSU sequence data and morphology, Crous et al. [7] established the genus *Rutola* within the Torulaceae. Later, Boonmee et al. [8] introduced *Cylindrotorula* within the Torulaceae with evidence from morphology and multi-gene phylogenetic analyses (ITS, LSU, RPB2, TEF). At present, the family Torulaceae includes genera *Cylindrotorula, Dendryphion*, *Neotorula*, *Rostriconidium*, *Rutola*, *Sporidesmioides*, and *Torula* [1,2,5,6,7,8].

The genus *Torula* was initially described by Persoon [9]. Conidiogenesis in *Torula* was observed by Crane and Schoknecht [10] using light and transmission electron microscopy. The phylogenetic relationships of the Torulaceae (Pleosporales) were studied by Crous et al. [1], and *Torula* was accepted within the family. Additionally, they identified three new species based on the number of septa per conidium and designated a neotype (CBS H-22275) for the generic type, *Torula herbarum* [1]. *Torula* has an ancient origin and has 541 species epithets in Index Fungorum (http://www.indexfungorum.org/; accessed on 20 January 2023). However, most species of *Torula* were introduced before the arrival of Sanger sequencing; therefore, there are a few molecular data available. *Torula* species are commonly collected from dead branches and submerged wood in terrestrial or freshwater habitats [4,5]. The genus is characterized by terminal or lateral, monoblastic or polyblastic conidiogenous cells with a basally thickened and heavily melanized wall and a thin-walled apex, frequently collapsing and becoming coronate [1,4,11]. This genus is a mold that lacks a known sexual state. The different species of *Torula* can be distinguished by conidial size and the number of septa and chains [1,4]. Recently, some species were introduced based on both molecular data and morphology [5,12,13,14]. 

We are studying the diversity of fungi in southwestern China, Sichuan, along the Yangtze River, and surveying the taxonomy of hyphomycetes [15,16,17,18,19]. During the survey of torula-like species in Chengdu Province (July to September 2021), nine isolates were obtained from wood-based substrates. Based on the in-depth phylogenetic analysis of combined (ITS, LSU, SSU, RPB2 and TEF) sequence data and morphological examination, these isolates were identified as three known *Torula* species, including a new geographic record (*T. masonii*) and four new species, viz., *T. chinensis* sp. nov., *T. submersa* sp. nov., *T. sichuanensis* sp. nov. and *T. longiconidiophora* sp. nov., which are introduced herein.

## 2. Materials and Methods

### 2.1. Sample Collection, Isolation and Morphological Examination

A survey on the diversity of hyphomycetes in Sichuan, China, was conducted between July and September of 2021. Dead branches of unknown hosts were collected from 5 locations (Yunqiao Wetland, Chengdu City; Baiyungou, Chongzhou City; Huilonggou, Pengzhou City; Hongfengling, Deyang City; Longchang, Neijiang City) in Sichuan. The specimens were taken into the laboratory in paper envelopes for examination. Microscopic characteristics were observed and recorded using a Nikon SMZ800N stereo microscope equipped with a Nikon DS-Fi3 microscope camera and a Nikon ECLIPSE Ni-U microscope fitted with a Nikon DS-Ri2 microscope camera, respectively. Measurements were conducted using the Nikon NIS-Elements Documentation Imaging Software Version 5.21.00. All photos were processed using Adobe Photoshop software version 22.0. Single conidium isolation was performed following the method described by Senanayake et al. [20]. Germinated conidia were individually transferred to potato dextrose agar (PDA) media plates and incubated in the dark at 25 °C. Culture characteristics were examined and recorded after one week and at regular intervals.

Herbarium specimens were deposited in the Herbarium of Cryptogams Kunming Institute of Botany Academia Sinica (HKAS), Kunming, China; and the Herbarium of the University of Electronic Science and Technology (HUEST), Chengdu, China. The living cultures were deposited in the China General Microbiological Culture Collection Center (CGMCC) in Beijing, China, and the University of Electronic Science and Technology Culture Collection (UESTCC) in Chengdu, China.

### 2.2. DNA Extraction, PCR Amplification and Sequencing

Fungal genomic DNA was extracted from mycelia using Trelief^TM^ Plant Genomic DNA Kit (TSINGKE Biotech, Shanghai, P.R. China) according to the manufacturer’s protocol. Five partial loci, including the nuclear ribosomal internal transcribed spacer (ITS: ITS1-5.8S-ITS2), the nuclear ribosomal small subunit rRNA gene (SSU), the nuclear ribosomal large subunit rRNA gene (LSU), the translation elongation factor 1-alpha gene (TEF) and the second largest subunit of RNA polymerase II gene (RPB2) were amplified by polymerase chain reaction (PCR). The corresponding primer pairs and PCR conditions are listed in Table 1. The final reaction volume of the PCR reagent was 25 µL containing 2 µL of DNA template, 1 µL each of the forward and reverse primer, 8.5 µL of double-distilled water (ddH_2_O) and 12.5 µL of 2 × lash PCR MasterMix (mixture of DNA Polymerase, dNTPs, Mg^2+^ and optimized buffer; CoWin Biosciences, Jiangsu, China). The PCR products were visualized by 1% agarose gel electrophoresis. Sanger sequencing was conducted by Tsingke Biological Technology (Beijing, China).

### 2.3. Phylogenetic Analyses

According to the corresponding Sanger sequencing chromatograms, misleading data from the ends of raw sequencing fragments were manually trimmed and assembled into consensus sequences using SeqMan Pro version 7.1.0 (DNASTAR, Inc. Madison, WI, USA). Barcode sequences of all *Torula* species for which the sequence data are available and the outgroup taxon *Sporidesmioides thailandica* (MFLUCC 13-0840) were downloaded from the NCBI nucleotide database (Table 2) using the function read. GenBank data were integrated within the R package Analysis of Phylogenetics and Evolution (APE) [29].

The multiple sequence alignments were conducted using MAFFT [30] version 7.310 with options “--adjustdirectionaccurately --auto”, and the alignment files were further trimmed using trimAl version 1.4 [31] with the option “-gapthreshold 0.5”, which only allows 50% of taxa with a gap in each site. The best-fit nucleotide substitution models for each locus were selected using PartitionFinder version 2.1.1 [32] under the corrected Akaike information criterion (AICC). All sequence alignments were combined using an in-house Python script.

Maximum likelihood (ML) and Bayesian analysis were conducted based on individual and combined datasets. Five alignment datasets of SSU, ITS, LSU, TEF, and RPB2 were concatenated for multi-locus phylogenetic analysis. ML phylogenetic tree was obtained using the IQ-TREE version 2.0.3 [33], and the topology was evaluated using 1,000 ultrafast bootstrap replicates. The Bayesian analysis was conducted using parallel MrBayes version 3.2.7a [34]. Two different runs with 20 million generations and four chains were executed, and the initial 25% of sample trees were treated as burn-in. Tracer version 1.7.1 [35] was used to confirm that the MCMC runs reached convergence, and all ESS values were above 200. Then, the ML tree was annotated by TreeAnnotator version 2.6.4 implemented in BEAST [36] based on Mrbayes MCMC trees with no discard of burn-in and no posterior probability limit. The ML tree was visualized using ggtree [37] and further edited in Adobe Illustrator software version 16.0.0.

## 3. Results

### 3.1. Molecular Phylogeny

The combined dataset includes five loci (LSU: 1–846, ITS: 847–1397, SSU: 1398–2411, TEF: 2412–3278, RPB2: 3279–4161) from 32 strains of *Torula* and the outgroup taxon *Sporidesmioides thailandica* (MFLUCC 13-0840). The combined dataset is composed of 970 distinct patterns, 511 parsimony-informative sites, 411 singleton sites and 3239 constant sites. The best-fit evolution models were determined—GTR+I+G for the ITS, LSU and TEF partitions; K80+G for the SSU partition; and SYM+G for the RPB2 partition.

**Figure 1:**
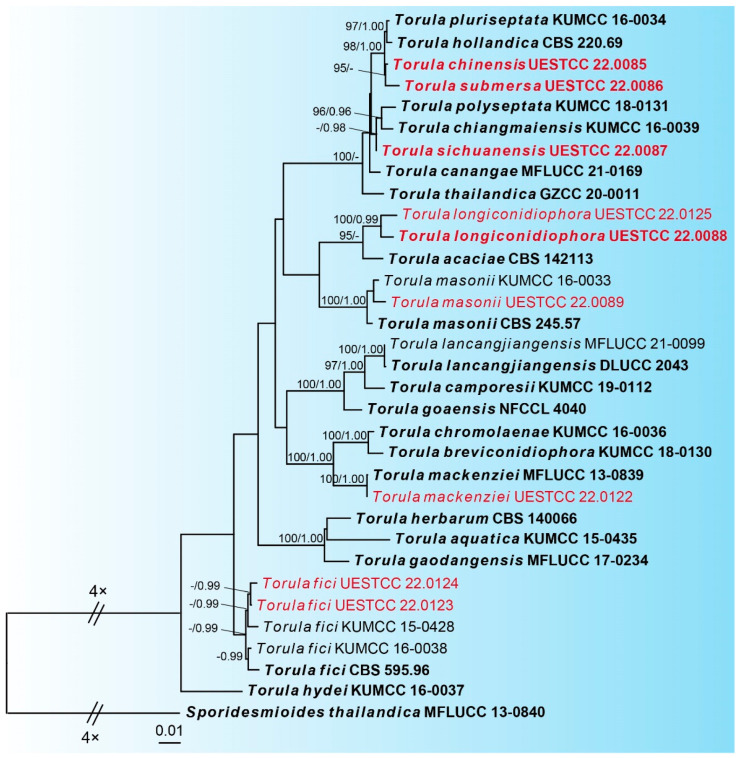
Phylogram of the best ML tree of species of *Torula* based on a combined dataset of LSU, ITS, SSU, TEF, and RPB2. Novel isolates are indicated in red. Type isolates are in bold. The ML ultrafast bootstrap values/Bayesian PP greater than 95%/0.95 are indicated at the respective nodes. The tree is rooted with *Sporidesmioides thailandica* (MFLUCC 13-0840).

The best-scoring ML tree (lnL = −14437.4141) with ultrafast bootstrap values from ML analyses and posterior probabilities from MrBayes analysis at the node is shown in Figure 1. Nine newly obtained *Torula* isolates represent seven different species. *Torula* isolate UESTCC 22.0089 is clustered with the ex-type stain of *T. masonii* (CBS 245.57). Our *Torula* isolate UESTCC 22.0122 and the ex-type stain *T. mackenziei* (MFLUCC 13-0839) are grouped into a statistically well-supported clade (100% ML, 1.00 BYPP). Two *Torula* isolates (UESTCC 22.0123 and UESTCC 22.0124) are clustered with the three *T. fici* strains, including the ex-type (CBS 595.96). The new species *Torula longiconidiophora* (UESTCC 22.0088 and UESTCC 22.0125) is separate from *T. acacia* (CBS 142113) with 95% ML support. The new species *Torula chinensis* (UESTCC 22.0085) is a sister to another new species, *Torula submersa* (UESTCC 22.0086), with 95% ML support. *Torula sichuanensis* (UESTCC 22.0087) formed a subclade with significant statistical support (91% ML/0.98 BYPP) and is sister to *T. polyseptata* (KUMCC 18-0131) and *T. chiangmaiensis* (KUMCC 16-0039).

### 3.2. Taxonomy

**Figure 2:**
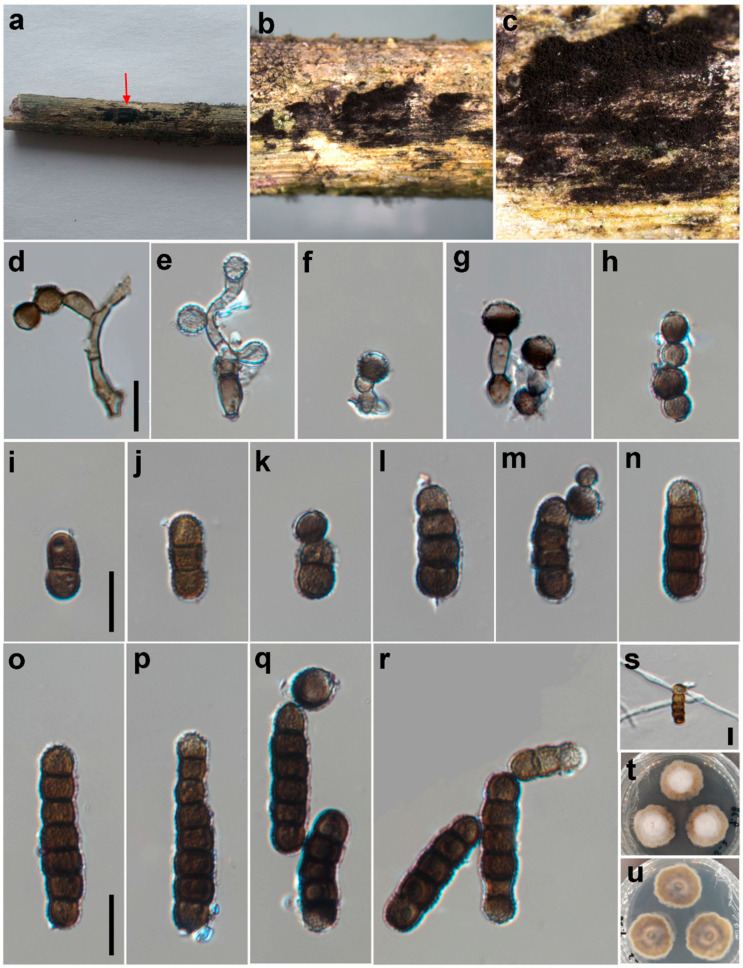
*Torula chinensis* (HKAS 126509, holotype). (**a**–**c**) Colonies on the natural substrate, arrows indicate the locations of colonies. (**d**–**h**) Conidiophores, conidiogenous cells and conidia. (**i**–**p**) Conidia. (**q**,**r**) Conidia in catenated chain. (**s**) Germinated conidium. (**t**,**u**) Culture on PDA from the surface and reverse. Scale bars: (**d**–**s**) = 10 μm. Scale bar of (**d**) applies to (**d**–**h**). Scale bar of (**i**) applies to (**i**–**n**). Scale bar of (**o**) applies to (**o**–**r**).

***Torula chinensis*** W.H. Tian, Y.P. Chen and Maharachch., sp. nov. (Figure 2).

*MycoBank*: MB 847014

*Etymology*: Named after the country, China, where it was collected.

*Saprobic* on decaying wood in a damp environment. **Asexual morph:** *Colonies* on natural substrate—effuse, black and powdery. *Mycelium* immersed or superficial, composed of septate, branched, dark brown to black hyphae. *Conidiophores* 2–4 μm wide, macronematous to semi-macronematous, septate, smooth, straight or slightly flexuous, dark brown, with 1–2 doliiform to globose cells. *Conidiogenous cells* 5–8 × 5–7 μm (*x* = 6.1 × 6.2, *n* = 25), polyblastic, terminal, dark brown to black, doliiform to subglobose, smooth to minutely verruculose and thick-walled. *Conidia* 6–37 × 4–8 μm (*x* = 21.5 × 6.0, *n* = 35), catenated, acrogenous, simple, phragmosporous, dark brown to black, minutely verruculose, 1–7-septate, rounded at both ends, composed of subglobose cells, often smaller at the apex, slightly constricted at some septa and chiefly subcylindrical. Conidial secession schizolytic.

*Material examined*: CHINA: Sichuan, Chongzhou City, Baiyungou, on a submerged decaying branch of an unknown host, N 30°47’56, E 103°24’15, elevation 990 m, 27 September 2021, W.H. Tian and Y.P. Chen BY45-2 (HKAS 126509, **holotype**), ex-type culture; CGMCC 3.24282 = UESTCC 22.0085.

*Culture characteristics*: Conidia germinated on PDA within 24 h at 25 °C. Colonies reached 32 mm after 10 days in an incubator under dark conditions at 25 °C. Colonies were raised in the middle; they were irregular circles; surface velvety; they had a white center and were yellowish-brown at the edges, with clear margins; reverse, yellowish brown with pale edges.

*Notes*: The phylogenetic tree shows that the isolate UESTCC 22.0085 is clustered with the ex-type strain of *Torula submersa* (UESTCC 22.0086) (Figure 1). *Torula chinensis* differs from *T. submersa* by having longer conidia (6–37 vs. 6–20 μm) and more septa in conidia (1–7 vs. 1–4). *Torula chinensis* resembles *T. pluriseptata* by having phragmosporous, minutely verruculose, subglobose cells and catenate conidia, but differs by having bigger conidiogenous cells (5–8 × 5–7 μm vs. 3.2–3.5 × 3.8–4.6) and fewer septa and shorter conidia (1–7-septa conidia 6–37 × 4–8 μm vs. 3–10-septa conidia 23.5–36 × 3.6–4.4 μm) [38]. Thus, considering the significant differences in morphology and molecular data, we describe the isolate UESTCC 22.0085 as *T. chinensis* sp. nov. 

**Figure 3:**
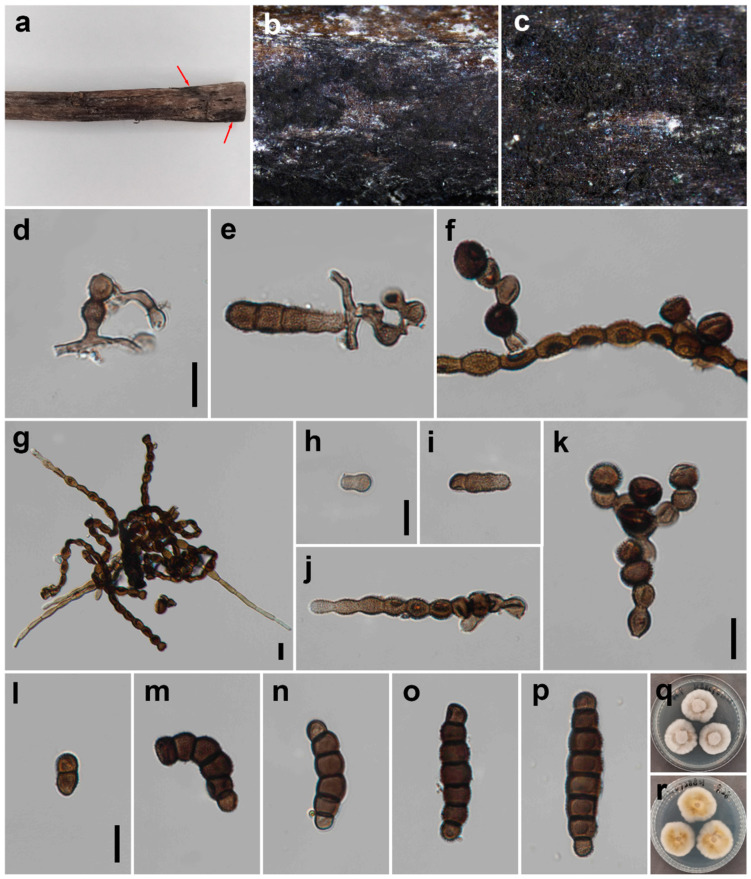
*Torula longiconidiophora* (HKAS 126512, holotype). (**a**–**c**) Colonies on the natural substrate; arrows indicate the locations of colonies. (**d**–**f**) Conidiophores with conidiogenous cells. (**g**) Mass of conidiophores. (**h**–**j**) Conidiophores. (**k**) Conidiogenous cells. (**l**–**p**) Conidia. (**q**,**r**) Culture on PDA from the surface and reverse. Scale bars: (**d**–**p**) = 10 μm. Scale bar of (**d**) applies to (**d**–**f**). Scale bar of (**h**) applies to (**h**–**j**). Scale bar of (**l**) applies to (**l**–**p**).

***Torula longiconidiophora*** W.H. Tian, Y.P. Chen and Maharachch., sp. nov. (Figure 3).

*MycoBank*: MB 847015

*Etymology*: Referring to the longer conidiophores.

*Saprobic* on decaying wood in a damp environment. **Asexual morph:** *Colonies* effuse on the host, velvety, dark brown to black and powdery. *Mycelium* partly immersed, composed of septate, branched, smooth, dark brown to black hyphae. *Conidiophores* 9–166 × 2–6 μm (*x* = 74.1 × 5.3, *n* = 20), septate, solitary, straight or curly winded, light brown to dark brown, consisting of many subcylindrical to subglobose cells that are smooth to minutely verruculose, without apical branches and thick-walled, and some doliiform to globose cells, forming scars at the junction with the conidiogenous cells. *Conidiogenous cells* 6–12 × 6–11 μm (*x* = 7.2 × 8.3, *n* = 25), polyblastic, terminal or intercalary, produced branched chain; base cell slightly truncated, black, ellipsoid to coronal, smooth to verruculose and thick-walled. *Conidia* 12–46 × 6–11 μm (*x* = 36.6 × 9.4, *n* = 35), solitary to catenate, acrogenous, simple, phragmosporous, dark brown to black, minutely verruculose, 1–7-septate, rounded at both ends, composed of subglobose cells, wider cells in the middle, often smaller at apex, slightly constricted at some septa and chiefly subcylindrical. *Conidial secession* schizolytic.

*Material examined*: CHINA: Sichuan, Pengzhou City, Huilonggou, on decaying branch of unknown host, N 31°11′6, E 103°54′56, elevation 1400 m, 28 July 2021, Y.P. Chen HLG 0728821 (HKAS 126512, **holotype**), ex-type culture, CGMCC 3.24283 = UESTCC 22.0088.

*Culture characteristics*: Conidia germinating on PDA within 24 h at 25 °C. Colonies reaching 34 mm after 10 days in an incubator in the dark at 25 °C. Colonies raised in the middle, irregularly circular, surface velvety, with white centers and becoming light brown, with a white and clear margin; reverse, yellow to light brown in the center and white at the margin.

*Notes*: In an NCBI BLASTn search based on ITS sequences, the closest match of *T. longiconidiophora* was *T. acacia* (CBS 142113) with 97% sequence identity; they differ in 538/555 bp (3%) with two gaps [39]. In addition, multi-gene phylogenetic analysis of a combined dataset of the LSU, ITS, SSU, TEF and RPB2 showed that they are close relatives with 95% ML support (Figure 1). *Torula longiconidiophora* also differs from *T. acacia* in having significantly wider conidia (6–11 μm vs. 5–6 μm) and fewer septa (1–7 vs. 2–15). Furthermore, the conidial cells of *T. longiconidiophora* are wider in the middle and narrow at the ends, whereas the cells of *T. acacia* are more uniform [39]. *Torula longiconidiophora* differs from other species in the genus by having two types of conidiophores. Thus, considering the significant differences in morphology and molecular data, we describe the isolate UESTCC 22.0088 as *Torula longiconidiophora* sp. nov.

**Figure 4:**
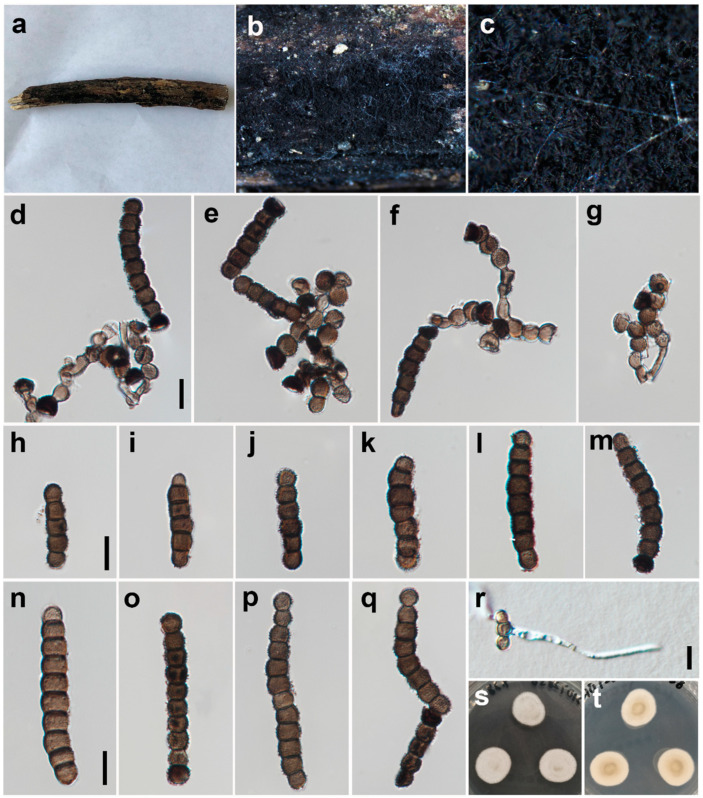
*Torula masonii* (HUEST 22.0090). (**a**–**c**) Colonies on the natural substrate. (**d**–**g**) Conidiophores, conidiogenous cells and conidia. (**h**–**q**) Conidia; (**r**) Germinated conidium. (**s**,**t**) Culture on PDA from surface and reverse. Scale bars: (**d**–**r**) = 10 μm. Scale bar of (**d**) applies to (**d**–**g**). Scale bar of (**h**) applies to (**h**–**m**). Scale bar of (**n**) applies to (**n**–**q**).

***Torula masonii*** P.W. Crous, IMA Fungus 6 (1): 192 (2015) (Figure 4).

*MycoBank*: MB 812806

*Saprobic* on decaying wood in a damp environment. **Asexual morph:** *Colonies* sparse, hairy, velvety, black on the substrate. *Mycelium* immersed or superficial, composed of septate, branched, dark brown to black hyphae. *Conidiophores* 2–4 μm wide, macronematous to semi-macronematous, septate, erect, straight or slightly flexuous, dark brown, subcylindrical to subglobose and thick-walled, with 1–2 doliiform to globose cells. *Conidiogenous cells* 4–7 × 5–8 μm (*x* = 5.5 × 6.5, *n* = 25), polyblastic, terminal or intercalary, dark brown to black, doliiform to subglobose, smooth to verruculose and thick-walled. *Conidia* 10–67 × 5–9 μm (?*x* = 35.2 × 6.5, *n* = 35), catenated, acrogenous, simple, phragmosporous, dark brown to black, minutely verruculose, 1–12-septate, rounded at both ends, composed of subglobose cells, often smaller at the apex (1–2 cells black at the apex), slightly constricted at some septa and chiefly subcylindrical. *Conidial secession* schizolytic.

*Material examined*: CHINA: Sichuan, Pengzhou City, Huilonggou, on decaying branch of unknown host, N 31°11′6, E 103°54′56, elevation 1400 m, 28 July 2021, Y.P. Chen HLG 072856 (HUEST 22.0090), living culture UESTCC 22.0089.

*Culture characteristics*: Conidia germinated on PDA within 24 h at 25 °C. The colony reached 21 mm after 10 days in an incubator in the dark at 25 °C. Colonies were white and irregularly circular with a velvety surface, with denser mycelium in the center and becoming sparser towards the edge, with clear margins; in reverse, pale green in the center and becoming white towards the edge.

*Notes*: *Torula masonii* was introduced by Crous et al. [1] based on the fungus sporulating in culture, which was collected from *Brassica* sp. in the UK. In this study, the phylogenetic tree shows that our isolate (UESTCC 22.0089) from decaying wood in a damp environment is clustered with the strain *T. masonii* (KUMCC 16-0033). In addition, isolate HUEST 22.0089 displays similar morphological characteristics with the type species of *T. masonii* (CBS 245.57) in colony shape, conidiophores, conidiogenous cells and conidia. We identified the isolate UESTCC 22.0089 as *T. masonii*, a new geographic record from a humid habitat in China.

**Figure 5:**
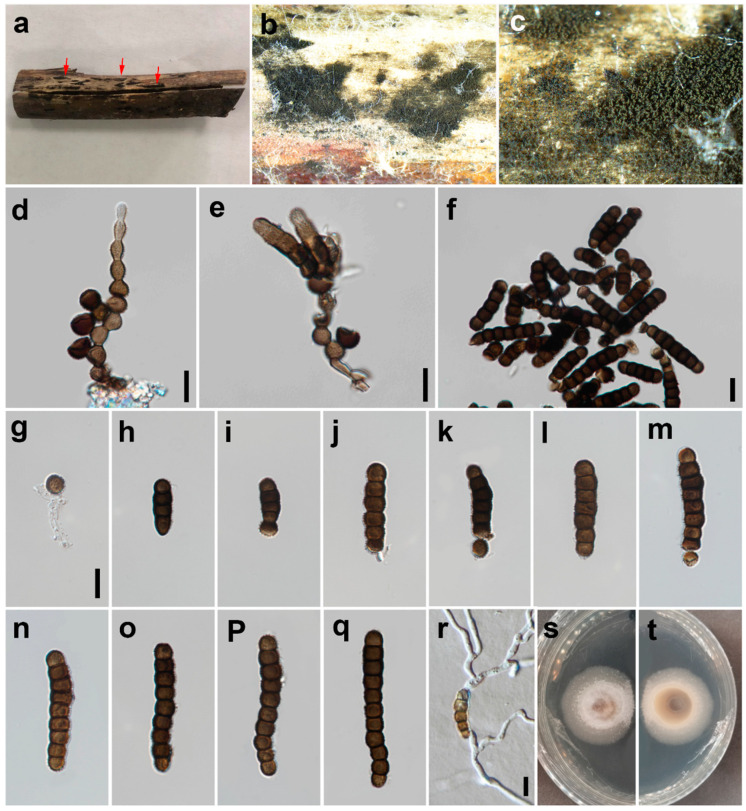
*Torula sichuanensis* (HKAS 126511, holotype). (**a**–**c**) Colonies on the natural substrate; arrows indicate the locations of colonies. (**d**,**e**) Conidiophores, conidiogenous cells and conidia. (**f**) Squash mount of partial colony. (**g**–**q**) Conidia; (**r**) Germinated conidium; (**s**,**t**) Culture on PDA from surface and reverse. Scale bars: (**d**–**r**) = 10 μm. Scale bar of (**g**) applies to (**g**–**q**).

***Torula sichuanensis*** W.H. Tian, Y.P. Chen and Maharachch., sp. nov. (Figure 5).

*MycoBank*: MB 847016

*Etymology*: Named after the province Sichuan, China, where it was collected.

*Saprobic* on decaying wood in a damp environment. **Asexual morph:** *Colonies* effuse on host, black and powdery. *Mycelium* partly immersed and composed of septate, branched, dark brown to black hyphae. *Conidiophores* 2–3 μm wide, macronematous to semi-macronematous, erect, solitary, thick-walled, brown, verruculose, consisting of 1–2 cells or reduced to conidiogenous cells and subcylindrical to subglobose. *Conidiogenous cells* 5–9 × 4–7 μm (*x* = 6.5 × 5.3, *n* = 25), polyblastic, terminal, dark brown to black, doliiform to subglobose, smooth to minutely verruculose and thick-walled. *Conidia* 7–80 × 5–7 μm (*x* = 31.3 × 6.3, *n* = 35), solitary to catenated, acrogenous, simple, phragmosporous, dark brown to black, minutely verruculose, 1–12-septate, rounded at both ends, composed of subglobose cells, often smaller at apex, slightly constricted at some septa and chiefly subcylindrical. *Conidial secession* schizolytic.

*Material examined*: CHINA: Sichuan, Deyang City, Yinghua town, Hongfengling, on submerged wood, N 31°20’56, E 103°59’47, elevation 1200 m, 16 October 2021, W.H. Tian and Y.P. Chen HFL16 (HKAS 126511, **holotype**), ex-type culture CGMCC 3.24284 = UESTCC 22.0087.

*Culture characteristics*: Conidia germinating on PDA within 24 h at 25 °C. Colonies reached 34 mm after 10 days in an incubator under dark conditions at 25 °C. Colony margins, regular; colonies have white aerial mycelia and a woolly center, with a sparse, thin layer and snowflake margin; reverse, brown in center and white in the margin.

*Notes*: Morphologically, *T. sichuanensis* is similar to *T. chiangmaiensis* and *T. pluriseptata*. However, they differ in the number of septa (1–12- epta vs. 4–12 septa and 3–10 septa) and the size of conidia (7–80 × 5–7 μm vs. 25.5–70 × 5.6–7.8 μm and 23.5–36 × 3.6–4.4 μm) [4]. The color of the conidia of *T. chiangmaiensis* is light brown to greyish-brown, *T. pluriseptata* is dark brown and *T. sichuanensis* is dark brown to black [4]. Phylogenetic analyses also confirmed they are distinct species (Figure 1). Therefore, we introduce the isolate UESTCC 22.0087 as a new species.

**Figure 6:**
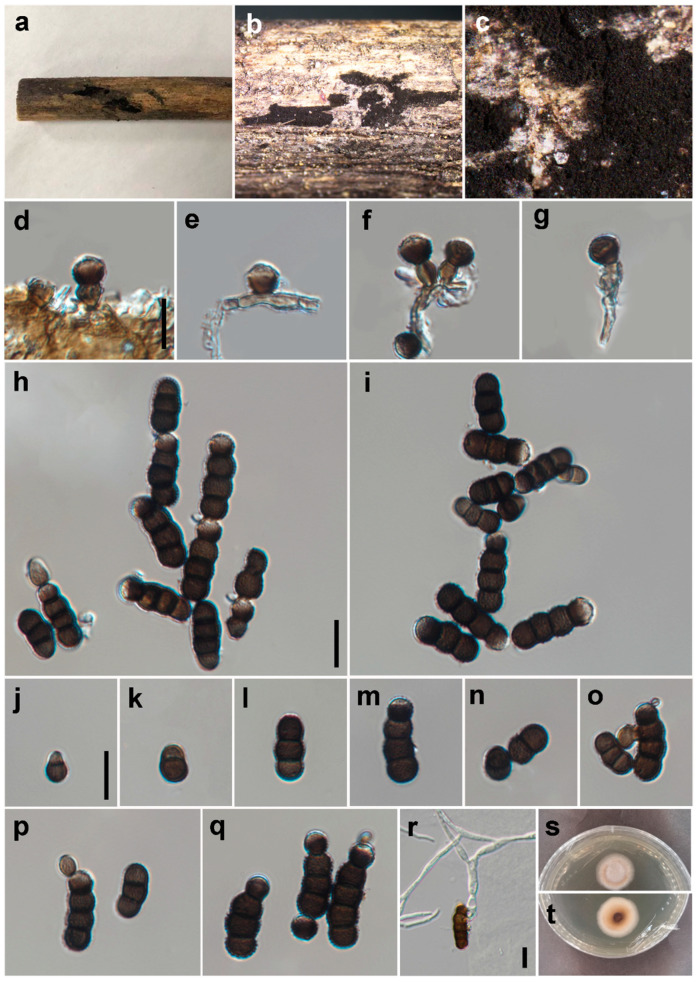
*Torula submersa* (HKAS 126510, holotype). (**a**–**c**) Colonies on the natural substrate; (**d**–**g**) Conidiophores with conidiogenous cells. (**h**,**i**) Branched chains of conidia. (**j**–**q**) Conidia. (**r**) Germinated conidium. (**s**,**t**) Culture on PDA from the surface and reverse. Scale bars: (**d**–**r**) = 10 μm. Scale bar of (**d**) applies to (**d**–**g**). Scale bar of (**h**) applies to (**h**,**i**). Scale bar of (**j**) applies to (**j**–**q**).

***Torula submersa*** W.H. Tian, Y.P. Chen and Maharachch., sp. nov. (Figure 6).

*MycoBank*: MB 847013

*Etymology*: Named referring to the submerged habitat of this fungus.

*Saprobic* on submerged decaying wood. **Asexual morph:** *Colonies* effuse, black, velvety and powdery on the host. *Mycelium* partly immersed and composed of septate, branched, black hyphae. *Conidiophores* 1–4 μm wide, macronematous to semi-macronematous, solitary, smooth, erect, thick-walled and dark brown, with one doliiform to globose cell. *Conidiogenous cells* 5–6 × 5–6 μm (*x* = 5.6 × 5.5, *n* = 25), monoblastic, terminal, dark brown to black, paler at apex, doliiform to subglobose, smooth to minutely verruculose and thick-walled. *Conidia* 6–20 × 4–8 μm (*x* = 14.5 × 6.0, *n* = 35), catenated, acrogenous, simple, phragmosporous, black, minutely verruculose, 1–4-septate, rounded at both ends, composed of subglobose cells, often paler at the apex, slightly constricted at some septa and chiefly subcylindrical. *Conidial secession* schizolytic. **Sexual morph:** Unknown.

*Material examined*: CHINA: Sichuan, Chongzhou City, Baiyungou, on a submerged decaying branch of unknown host, N 30°47’56, E 103°24’15, elevation 990 m, 27 September 2021, W.H. Tian and Y.P. Chen BY60-2 (HKAS 126510, **holotype**), ex-type culture CGMCC 3.24281 = UESTCC 22.0086.

*Culture characteristics*: Conidia germinated on PDA within 24 h at 25 °C. Colonies reached 26 mm after 10 days in an incubator in the dark at 25 °C. Colonies were raised in the middle, irregularly circular, velvety on the surface and had a white center fading to yellowish brown and white edges; they hadclear margins; reverse, yellowish brown in center with white margin.

*Notes*: The phylogenetic tree shows that the isolate UESTCC 22.0086 is clustered with the ex-type strain of *Torula chinensis* (UESTCC 22.0085) (Figure 1). Multigene phylogenetic analysis of a combined dataset of the LSU, ITS, SSU, TEF, and RPB2 showed that *T. chinensis* and *T. submersa* are close relatives with 95% ML support (Figure 1). Morphologically, *T. submersa* differs from other *Torula* species by having black conidia composed of subglobose cells and 1–4 septa (see notes under *T. chinensis*). Therefore, we introduce the isolate UESTCC 22.0086 as a new species.

## 4. Discussion

Even though over 541 epithets of *Torula* have been reported, previously, many *Torula* species were identified based on morphology alone, and there were very little data relating to the phylogenetic relationships until the study of Crous et al. [1]. Therefore, it is possible that a number of the species in *Torula* are conspecific or belong to different genera. Re-examining type specimens of *Torula*-like species described prior to the advent of molecular technology is necessary to address this issue. Additionally, fresh specimens should be collected, sequenced and combined with multi-locus phylogenetic analysis and morphological examination, and designation of epi-types is essential. For instance, Crane et al. [11] re-examined several hyphomycetous species previously placed in the *Torula* and found that *T. rhombica* and *T. terrestris* do not seem to be congenerous with *T. herbarum*, and transferred to them *Bahusandhika*. Recently, new species have been identified based on molecular data and morphology, and to date, only 23 species have sequence data [1,3,4,5,8,11,12,13,14,20,38,39,40]. 

The genus *Torula* contains many diverse species, frequently isolated from submerged decaying wood, living leaves, dead wood and twigs of various terrestrial plants, soil and earthworm casts [41]. They are commonly isolated as saprobes in temperate and tropical climate regions [42]. A few species are plant pathogens, such as *T. herbarum*, causing stem blight in Indian jujube (*Ziziphus mauritiana*) [43]. They are also isolated from the air of natural environments, parks and industrial zones and are reported to cause seasonal fungal allergies in humans [44,45,46,47,48]. The genus has been shown to produce a wide range of chemically novel diverse secondary metabolites. For example, *T. herbarum* displays antibacterial, antifungal, antiamoebic and potentially anti-cancer properties [49,50,51]. Therefore, more taxonomic, phylogenic and biochemical studies of this bioprospecting genus should be performed.

## Figures and Tables

**Table 1:** Loci used in this study with the corresponding PCR primers and conditions.

Locus	PCR Primers	PCR: Thermal Cycles	References
ITS	ITS9mun or ITS5/ITS4_KYO1 or ITS4	(94 °C: 30 s, 56 °C: 30 s, 72 °C: 30 s) × 35 cycles	[21,22]
LSU	LR0R/LR5	(94 °C: 30 s, 56 °C: 30 s, 72 °C: 1 min) × 35 cycles	[23,24]
SSU	PNS1/NS41	(94 °C: 30 s, 56 °C: 30 s, 72 °C: 1 min) × 35 cycles	[25]
TEF	EF1-983/EF1-2218R	(94 °C: 30 s, 52 °C: 30 s, 72 °C: 1 min) × 35 cycles	[26,27]
RPB2	dRPB2-5f/dRPB2-7r	(94 °C: 30 s, 52 °C: 30 s, 72 °C: 1 min) × 35 cycles	[28]

**Table 2:** Species details and their GenBank accession numbers used in phylogenetic analyses. Type/epitype strains are in bold, and newly generated sequences are in red.

Species	Strain/Voucher No.	GenBank Accession Numbers				
		ITS	LSU	SSU	TEF	RPB2
***Sporidesmioides thailandica***	**MFLUCC 13-0840**	**MN061347**	**NG_059703**	**NG_061242**	**KX437766**	**KX437761**
***Torula acaciae***	**CBS 142113**	**NR_155944**	**NG_059764**	**-**	**-**	**KY173594**
***T. aquatica***	**KUMCC 15-0435**	**MG208167**	**MG208146**	**-**	**-**	**MG207977**
***T. breviconidiophora***	**KUMCC 18-0130**	**MK071670**	**MK071672**	**MK071697**	**MK077673**	**-**
***T. camporesii***	**KUMCC 19-0112**	**MN507400**	**MN507402**	**MN507401**	**MN507403**	**MN507404**
***T. canangae***	**MFLUCC 21-0169**	**OL966950**	**OL830816**	**-**	**ON032379**	**-**
***T. chiangmaiensis***	**KUMCC 16-0039**	**MN061342**	**KY197856**	**KY197863**	**KY197876**	**-**
***T. chinensis***	**UESTCC 22.0085**	**OQ127986**	**OQ128004**	**OQ127995**	**-**	**-**
***T. chromolaenae***	**KUMCC 16-0036**	**MN061345**	**KY197860**	**KY197867**	**KY197880**	**KY197873**
***T. fici***	**CBS 595.96**	**KF443408**	**KF443385**	**KF443387**	**KF443402**	**KF443395**
*T. fici*	KUMCC 15-0428	MG208172	MG208151	-	MG207999	MG207981
*T. fici*	KUMCC 16-0038	MN061341	KY197859	KY197866	KY197879	KY197872
*T. fici*	UESTCC 22.0123	OQ127978	OQ127996	OQ127987	OQ158972	OQ158969
*T. fici*	UESTCC 22.0124	OQ127979	OQ127997	OQ127988	OQ158972	OQ158970
***T. gaodangensis***	**MFLUCC 17-0234**	**MF034135**	**NG_059827**	**NG_063641**	**-**	**-**
***T. goaensis***	**NFCCL 4040**	**NR_159045**	**NG_060016**	**-**	**-**	**-**
***T. herbarum***	**CBS 140066**	**KR873260**	**KR873288**	**-**	**-**	**-**
***T. hollandica***	**CBS 220.69**	**NR_132893**	**NG_064274**	**KF443389**	**KF443401**	**KF443393**
***T. hydei***	**KUMCC 16-0037**	**MN061346**	**MH253926**	**MH253928**	**MH253930**	**-**
***T. lancangjiangensis***	**DLUCC 2043**	**MW723059**	**MW879526**	**MW774582**	**MW729785**	**MW729780**
*T. lancangjiangensis*	MFLUCC 21-0099	MZ538529	MZ538563	-	MZ567104	-
***T. longiconidiophora***	**UESTCC 22.0088**	**OQ127983**	**OQ128001**	**OQ127992**	**OQ158972**	**OQ158967**
*T.longiconidiophora*	UESTCC 22.0125	OQ127984	OQ128002	OQ127993	OQ158972	OQ158972
***T. mackenziei***	**MFLUCC 13-0839**	**MN061344**	**KY197861**	**KY197868**	**KY197881**	**KY197874**
*T. mackenziei*	UESTCC 22.0122	OQ127980	OQ127998	OQ127989	OQ158972	OQ158971
***T. masonii***	**CBS 245.57**	**NR_145193**	**NG_058185**	**-**	**-**	**-**
*T. masonii*	KUMCC 16-0033	MN061339	KY197857	KY197864	KY197877	KY197870
*T. masonii*	UESTCC 22.0089	OQ127982	OQ128000	OQ127991	-	-
***T. pluriseptata***	**KUMCC 16-0034**	**MN061338**	**KY197855**	**KY197862**	**KY197875**	**KY197869**
***T. polyseptata***	**KUMCC 18-0131**	**MK071671**	**MK071673**	**MK071698**	**MK077674**	**-**
***T. sichuanensis***	**UESTCC 22.0087**	**OQ127981**	**OQ127999**	**OQ127990**	**-**	**-**
***T. submersa***	**UESTCC 22.0086**	**OQ127985**	**OQ128003**	**OQ127994**	**OQ158972**	**OQ158968**
***T. thailandica***	**GZCC 20-0011**	**MN907426**	**MN907428**	**MN907427**	**-**	**-**

## Data Availability

All sequence data are available in NCBI GenBank with the accession numbers in the manuscript.
